# A proposed insight into the anti-viral potential of metallic nanoparticles against novel coronavirus disease-19 (COVID-19)

**DOI:** 10.1186/s42269-021-00487-0

**Published:** 2021-02-05

**Authors:** Ghadha Ibrahim Fouad

**Affiliations:** Department of Therapeutic Chemistry, National Research Centre, 33 El-Bohouth St., Dokki, Cairo, 12622 Egypt

**Keywords:** Coronaviruses, Nanotechnology, Metallic nanoparticles, Nanotheranostics, Anti-viral, COVID-19

## Abstract

**Background:**

Over the last ten months since December 2019, the world has faced infectious emerging novel coronavirus disease-2019 (COVID-19) outbreaks that had a massive global impact affecting over 185 countries.

**Main body:**

Emerging novel COVID-19 is a global health emergency on a pandemic scale that represents a terror to human health through its ability to escape anti-viral measures. Such viral infections impose a great socioeconomic burden, besides global health challenges. This imposes a pressing need for the development of anti-viral therapeutic agents and diagnostic tools that demonstrate multifunctional, target-specific, and non-toxic properties. Nanotheranostics is regarded as a promising approach for the management of different viral infections. Nanotheranostics facilitates targeted drug-delivery of anti-viral therapeutics as well as contributing to the development of diagnostic systems. Multifunctional metallic nanoparticles (NPs) have emerged as innovative theranostic agents that enable sustainable treatment and effective diagnosis. Here we have reviewed current advances in the use of theranostic metallic NPs to fight against COVID-19, and discussed the application as well as limitations associated with nanotechnology-based theranostic approaches.

**Conclusion:**

This review verified the potential use of some metal-based NPs as anti-viral nanotheranostic agents. Metal-based NPs could act as carriers that enable the sustainable and targeted delivery of active anti-viral molecules, or as diagnostic agents that allow rapid and sensitive diagnosis of viral infections.

## Background

Viral infections are one of the principal causes of mortality in the entire world that result in significant human, social, and economic costs (Morens et al. 2004). The elevated possibility of incidence of the viral outbreak might be ascribed to the limitations in the applied detection systems as well as the adaptive nature of viruses with large genomes (Frenk et al. 2011). Therefore, the implementation of highly sensitive and specific diagnostic tools is an urgent demand to detect viral infections and to manage or control viral spreading. In the current outbreak of COVID-19, the scientists expected that the world would face a resurgence of COVID-19 in lockdowns regions in China (Cyranoski 2020).

Nowadays, the outbreak of the highly infectious novel COVID-19 started in a local market in Hubei province in Wuhan city, China in December 2019, and then expanded globally to affect hundreds of countries. A joint World Health Organization (WHO)-China report stated that the pandemic surged between late January and early February 2020 (WHO 2020c, d). By 11 March 2020, WHO declared this outbreak of coronavirus-associated pneumonia as a global pandemic.

United States of America, India, and Brazil have emerged as centers of COVID-19 outbreak. The total confirmed global cases of COVID-19 have reached to about 34, 947, 761 until 4 October 2020. The USA comes in the first place by (7, 433, 828), followed by India (6, 623, 815), and Brazil (4, 915, 289). In addition, COVID-19 hit several countries in the Middle East, such as Iraq (382, 949), Kingdom of Saudi Arabia (336, 766), Morocco (133, 272), Qatar (126, 692), Kuwait (107, 592), and Egypt (103, 686). This viral infection results in a huge toll of death exceeding 1, 038, 797. COVID-19 Dashboard by the Center for Systems Science and Engineering (CSSE) at Johns Hopkins University (JHU) that provided these numbers of cumulative confirmed cases (CSSE 2020).

Since 2012, WHO raised a global alert for a new severe acute respiratory syndrome (SARS)-like coronavirus, which is now called Middle East Respiratory Syndrome (MERS) coronavirus. MERS is one of the most prominent viral infections in the Middle East region that caused thousands of infections and toll of deaths in a short time (Draz and Shafiee 2018). The novel pneumonia-causing COVID-19 is a rapidly spreading and communicable viral infection that can cause pneumonia-associated mortality in about 2.5% of infected cases; this pathogen is closely related to sever acute respiratory syndrome CoV (SARS-CoV) (Wang et al. 2020a).

To face this current outbreak of COVID-19, WHO rapidly provided seven RT-PCR assays for detection of viral RNA of SARS-CoV-2, through converting it to complementary DNA (cDNA) that is subsequently amplified and detected using fluorescence probes (WHO 2020e).

There is a global demand to improve and validate “point-of-care (POC) Diagnostics” of Viral RNA for COVID-19 through establishing large-scale, rapid, cost-effective and simple diagnostic tools that could enable the signal amplification of the very low viral RNA loads in different specimens. These POC tests should enable the minimal exposure to the virus by the operators through performing the analysis in a closed environment, in a relatively short time (Feng et al. 2020). The main priority for COVID-19 diagnostics research is to develop nucleic acid and protein tests and detection at the POC that could be combined into “multiplex panels”. This POC-diagnostics research can enable a better evaluation of total SARS-CoV-2 infections through monitoring and tracking both infected and recovered COVID-19-patients (Udugama et al. 2020).

Therefore, it is a mandatory requirement to slow down the spread of the viral infections in the population to support healthcare systems to respond efficiently to severe COVID-19 cases as well as to non-COVID-19 health needs, as demonstrated in recent guidance documents of the European Centre for Disease Prevention and Control (ECDC). Moreover, countries should enhance their COVID- 19 diagnostic testing capacity in healthcare systems (ECDC 2020). The recommended diagnostic test is "viral RNA detection" using Real-time polymerase chain reaction (RT-PCR) (WHO 2020c, d). WHO does not recommend the use of rapid tests based on antigen testing or antibody detection, due to accuracy issues in the absence of validation studies; the sensitivity of antigen tests might be expected to vary from 34 to 80%, while for antibody tests, the majority of patients develop antibody response only in the second week after onset of symptoms. Antibody detection tests targeting COVID-19 may also cross-react with other pathogens, including other human coronaviruses and give false-positive results (WHO 2020e). The aim of this review is to present a comprehensive discussion of the potential use of the metallic nanoparticles as nanotherapeutics against the current pandemic of SARS-COV-2.

## Research methodology

### Identification of a framework and definition of the hypothesis

In this review, we proposed a frxsamework for understanding the possible link between the current pandemic of SARS-COV-2 and metallic nanoparticles (NPs). The working hypothesis of this review was that NPs are expected to act as anti-viral therapeutic agents against COVID-19 or as diagnostic agents that contribute to the detection of viral particles of SARS-COV-2. Therefore, this framework aimed to collect the most recent and relevant studies that examined the retrieved assumptions.

### Search strategy

This review included studies from several databases such as “SCOPUS, Science Direct, PubMed, and Web of Science” in addition to sites related to the current topic such as that of the World Health Organization (WHO). The search strategy was based on using search terms (in combination) including “coronavirus”, “COVID-19”, “nanoparticles”, “antiviral”, and “silver nanoparticles”, “gold nanoparticles”, “copper nanoparticles”, and “titanium nanoparticles”. This review covered over 70 papers and demonstrated the nanotheranostics application of metallic NPs as a possible strategy for the management of the viral infections. The reference lists of selected articles were checked manually, to obtain additional eligible studies that not retrieved in the databases.

### Inclusion and exclusion criteria

The identified framework and review question determine the search methodology and the selection criteria, both inclusion and exclusion criteria. The inclusion criteria were: (1) the anti-viral potential of metal-based NPs, (2) the nanotheranostic agents, (3) English-language study, only research articles, and review articles, which were published in a peer-reviewed scientific journal, in the period between 2010 and 2020. (4) The main subject areas involve "Biochemistry, Genetics and Molecular Biology, Pharmacology, Toxicology and Pharmaceutics, Immunology and Microbiology, and multidisciplinary". The exclusion criteria includes “organic”, “bacterial”, “synthesis”, and “Chemistry”. Exclusion by the subject area included chemistry, material science, medicine, chemical engineering, engineering, physics, computer science, and neuroscience. We had excluded the conference abstracts, book chapters, editorials, short communications, and papers without full text. This review included studies that focused on the antiviral potential of metallic NPs.

### Data extraction and definitions

Data extraction from the included studies was done using a data collection table (Table 1) containing significant information related to each article including the following variables: chemical composition, morphological features (size, and shape), the characterization method, application, function, and references.

**Table 1:** Data-collection table demonstrated the anti-viral potential of metallic NPs for viral inhibition and detection

Chemical composition and size (nm) of NPs	Characterization method	Virus	Application and function	References
Gold NPs (AuNPs) (15 nm)	Data not shown	Hepatitis C virus (HCV)	Gold (Au) NP probes (GNP) for ultrasensitive detection of HCV core antigen (HCVcAg)	Yin et al. (2017)
Gold NPs (AuNPs) (30 nm) and Fe_3_O_4_ NPs (15–20 nm)	SEM,TEM	Hepatitis B virus (HBV) surface antigen	Immunosensor for surface antigen detection based on Hemin/G-quadruplex horseradish peroxidase-mimicking DNAzyme-signal amplification	Alizadeh et al. (2017)
Gold NPs (AuNPs) (15 ± 4 nm)	Data not shown	Hepatitis B virus (HBV) surface antigen	DNA/AuNPs sensor: a sandwich assay and surface-enhanced Raman scattering (SERS) sensor for ultrasensitive detection of viral DNA	Zengin et al. (2017)
Gold NPs (AuNPs) (2 nm and 14 nm)	TEM	Influenza virus A (H1N1)	Multivalent Sialic-Acid-Functionalized AuNPs: anti-viral activity	Papp et al. (2010)
Gold NPs (AuNPs) (35 nm)	Filter-based multimode microplate reader, TEM	Influenza virus A (H1N1)	A modified enzyme-linked immunosorbent assay (ELISA) with nanomaterials: anti-viral activity	Ahmed et al. (2016)
Gold NPs (AuNPs) (2–50 nm)	SEM	Hepatitis C virus	For direct detection of unamplified viral RNA in clinical specimens	Shawky et al. (2010)
Gold NPs (AuNPs) (19 nm)	TEM, DLS	Middle East respiratory syndrome coronavirus (MERS-CoV)	Detection method of MERS-CoV with a potential detection limit of 1 pmol/μL	Kim et al. (2019)
Gold NPs (AuNPs) (40 and 100 nm)	DLS and TEM	Severe acute respiratory syndrome coronavirus (SARS‐CoV)	AuNPs ‐adjuvanted S protein: to induce immune response (IgG)	Sekimukai et al. (2019)
Gold NPs (AuNPs) (5–20 nm)	XRD, SEM, AFM	Adenoviruses	AuNPs encapsulated in a SiO_2_ shell: Anti-viral activity	Lysenko et al. (2018)
Gold NPs (AuNPs) (12 nm)	TEM, UV–Vis spectrophotometry, DLS	Influenza A virus	AuNPs—matrix 2 protein (M2e) conjugate coformulated with CpG: to induce immune response	Tao et al. (2014)
Gold NPs (AuNPs) (18 nm)	CLSM	Influenza virus	AuNPs-based vaccination to enhance adaptive immunity	Wang et al. (2018)
Gold NPs (AuNPs)	UV–Vis spectroscopy, TEM	Bovine viral diarrhea virus (BVDV)	Peptide nucleic acids (PNA)-AuNPs colorimetric detection assay of viral RNA	Askaravi et al. (2017)
Gold NPs (AuNPs) (13 nm)	TEM, DLS	Influenza A virus	A colorimetric immunosensor based on AuNPs modified with monoclonal anti-hemagglutinin antibody (mAb)	Liu et al. (2015)
Gold NPs (AuNPs) (10, 20, and 40 nm)	SEM	Influenza A virus	Immuno-AuNPs conjugates: for detection	Gopinath et al. (2013)
Gold NPs (AuNPs) (12.92 nm)	UV–Vis spectroscopy, DLS	Dengue virus	Cationic AuNPs–siRNA complexes: in vitro anti-viral activity	Paul et al. (2014)
Silver NPs (AgNPs) (30–50 nm)	Data not shown	Human immunodeficiency virus 1 (HIV-1)	Therapeutic (anti-viral) action at an early stage and at post-entry stages	Lara et al. (2010)
Silver NPs (AgNPs) (13, 33 and 46 nm)	TEM, SEM	Herpesviruses (HSV-2) infectivity	Tnnic acid modified AgNPs: Size-related in vitro and in vivo anti-viral activity	Orłowski et al. (2018)
Silver NPs (AgNPs) (33 nm)	TEM	Herpes simplex virus (HSV) type 1 and 2	Tannic Acid/ AgNPs-Based Mucoadhesive Hydrogel: anti-viral activity	Szymańska et al. (2018)
Silver NPs (AgNPs) (5- 25 nm)	High-resolution transmission and field-emission scanning electron microscopes	Non-Enveloped (feline coronavirus (FCoV)) and Enveloped (bursal disease virus (IBDV)) Viruses	Graphene–Silver Nanocomposites: anti-viral activity	Chen et al. (2016)
Silver NPs (AgNPs) (10 nm)	TEM	Influenza virus A (H1N1)	Anti-viral activity	Xiang et al. (2011)
Silver NPs (AgNPs) (3.5, 6.5, and 12.9 nm)	SEM and TEM	Influenza virus A (H1N1)	AgNPs/chitosan (Ch) composites: anti-viral activity	Mori et al. (2013)
Silver NPs (AgNPs) (7–20 nm)	UV–Vis spectrophotometry, Nanoparticle tracking and analysis, TEM	Herpes simplex virus and human parainfluenza virus type 3	Smaller-sized AgNPs: anti-viral activity through decreasing viral replication	Gaikwad et al. (2013)
Silver NPs (AgNPs) (4–9 nm)	UV–Vis spectrometry, TEM, EDX- FE-SEM	Poliovirus and non-enveloped viruses	Electrochemical –synthesized AgNPs: anti-viral activity	Huy et al. (2017)
Silver NPs (AgNPs) (11.4 nm)	TEM	Adenovirus type 3 (Ad3)	In vitro anti-viral activity	Chen et al. (2013)
Silica NPs (SiNPs) (200 nm)	TEM, SEM	Porcine circovirus type 2 (PCV2)	Hollow mesoporous silica nanoparticles (HMSNs): to act as a protein delivery system or vaccine carriers	Guo et al. (2012)
Silica NPs (SiNPs) (50–70 nm)	FESEM, FTIR, TGA	Human immunodeficiency virus (HIV)	Silica NPs-based Immunoassay (SNIA): for detection using time resolved fluorescence- enhanced sensitivity	Chunduri et al. (2017)
Silica NPs (SiNPs) (150–200 nm)	DLS, TEM	Herpes simplex virus (HSV) infections	Glycosaminoglycans (GAG) mimetic functionalised solid and mesoporous SiNPs (MSNs and SSNs): acting as viral binding/entry inhibitors	Lee et al. (2016)
Iron oxide NPs (IONPs) (10–15 nm)	HR-TEM, DLS-zeta, XRD, FTIR	Pandemic influenza strain A/H1N1/Eastern India/66/PR8-H1N1	Inhibition of virus growth	Kumar et al. (2019)
Iron oxide magnetic NPs (MNPs) (90 ± 30 nm)	TEM, XRD	Influenza A virus	An assay combining efficient magnetic separation and MALDI-TOF MS: For detection	Chou et al. (2011)
Iron oxide NPs (IONPs) (103 ± 2 nm)	Malvern’s zetasizer-ZS90	Zika Virus	Magnetic relaxation-sensitive nanoparticles (MRNPs): Magnetic Nanosensors for detection	Shelby et al. (2017)
Zinc oxide NPs (ZnONPs) (20–50 nm)	ICP-MS, XRD, TEM, FE-SEM	Influenza virus A (H1N1)	Anti-viral activity	Ghaffari et al. (2019)
Zinc oxide (ZnO)-nanostructures (diamters:200 nm to 1 µm; arm lengths:5 µm to 30 µm)	SEM	Herpes simplex virus type-2 (HSV-2)	ZnO tetrapod micro-nanostructures synthesized by flame transport approach: anti-viral activity	Antoine et al. (2012)
Graphene oxide (GO) (127.7 nm)	UV–vis spectroscopy Raman spectroscopy	Respiratory syncytial virus (RSV)	Curcumin functionalized GO: anti-viral activity	Yang et al. (2017)
Titanium dioxide (TiO_2_) (4–10 nm)	EM	Influenza Virus	Anti-viral activity	Mazurkova et al. (2010)
Ceria NPs (6 nm)	TEM	Influenza virus	Nano-ceria aqueous sol containing no stabilizer: to enhance immune response	Zholobak et al. (2016)
Selenium NPs (SeNPs) Se@ZNV: 82 nm	TEM, EDX, FTIR, XPS	Influenza virus A (H1N1)	Zanamivir modified SeNPs (Se@ZNV): anti-viral activity	Lin et al. (2017)

**Table 2:** demonstrated the pros and cons about some standard anti-viral drugs used to treat SARS-CoV-2

Anti-viral drug	Pros	Cons
Chloroquine (CQ) and Hydroxychloroquine (HCQ) (Anti-malarial drugs)	Both are safe anti-inflammatory agents that effectively inhibit the viral entry (Liu et al. 2020) Both are associated with efficient absorption in different tissues (Liu et al. 2020) HCQ is more efficient than CQ, based on molecular docking analysis (Achutha et al. 2020)	CQ and HCQ may interfere the functioning of other drugs or at high doses (Pal et al. 2020; Zou et al. 2020) HCQ is associated with cardiotoxic effects (e.g. drug-induced cardiomyopathy) and gastrointestinal effects (Pal et al. 2020)
Tocilizumab (TCZ) (IL-6 receptor (IL-6R) antagonist)	TCZ act by inhibiting IL-6 binding to receptors and decreasing cytokine storm (Zhanga et al. 2020) There were no TCZ -linked complications or illness deterioration (Zhanga et al. 2020)	TCZ increases the risk of cardiovascular disease (Zhanga et al. 2020) TCZ might cause some adverse effects including hepatic damage, neutropenia, thrombocytopenia, or serious secondary infection (Zhangb et al. 2020; Kotak et al. 2020)
Remdesivir (RDV) (A prodrug of an adenosine analogue)	RDV is a broad-spectrum antiviral agent and a potent inhibitor of SARS-CoV-2 replication in nasal and bronchial airway epithelial cells (Doggrell 2020) RDV decreases the recovery-time in COVID-19 patients and currently is the first approved anti-viral drug against COVID-19 (Beigel et al. 2020)	RDV might cause some side effects such as the elevation in hepatic enzyme levels and diarrhea (Grein et al. 2020) The poor oral-bioavailability of RDV hindered its prophylactic use (Malin et al. 2020)
Lopinavir/ritonavir (LPVr) (a protease inhibitor)	LPV/r plays an important role in the “early stage i.e. initial 7–10 days” clinical outcome (Yao et al. 2020) LPV/RTV-treated COVID-19 patients demonstrated a lower mortality rate than HCQ (Karolyi et al. 2020)	LPV might cause some adverse reactions including elevated hepatic enzymes, increased triglycerides, in addition to, diarrhea, nausea, asthenia, and gastrointestinal side effects (Yao et al. 2020) LPVr could increase the risk of bradycardia (Beyls et al. 2020)
Ribavirin	Ribavirin is a broad-spectrum antiviral drug that is available in oral dosage forms (Wang and Zhu 2020)	Ribavirin demonstrated some adverse reactions including fever, headache, neutropenia, in addition to, hemolytic anemia (Wang and Zhu 2020) Ribavirin, at high doses, increases the risk of hematologic toxicity, besides, its poor in vitro efficacy and poor outcomes. Therefore, ribavirin was not considered a viable treatment for further investigation by the WHO in COVID-19 patients (McCreary and Pogue 2020)

## Results

The framework of this review is a multi-step process including; defining the problem, developing the search strategy, identifying relevant publications, evaluating the retrieved documents, extracting and collecting evidence-based data, organizing and integrating data into an evidence-based review article. Seventy relevant publications, in the period between 2010 and 2020, were analyzed and studied. Of these publications, 39 were original research papers and 31 were review papers. For each retrieved publication, the information was collected in a data extraction table (Table 1).

## Discussion

The current review relies on the potential application of metallic NPs as a therapeutic or diagnostic tool to control the spread of the viral infection of SARS-COV-2, through presenting evidence-based and transparent solutions extracted from previous studies. Because of the urgent need for novel anti-viral agents on the current and future emerging pandemic states of SARS-COV-2, this review suggests the use of nanotheranostics to take part in the management (treatment and detecting) of COVID-19 infections through providing evidence-based data that promote the field progression toward establishing anti-viral nanotheranostics to combat COVID-19.

### The research question: coronaviruses (CoVs) and current limitations

Coronaviruses (CoVs) are large enveloped RNA viruses that are pleomorphic in shape; CoVs have a large positive-sense, single-stranded RNA genome of 27–32 kb, which encodes four to five structural proteins and two non-structural polyprotein precursors (Perlman and Netland 2009). This family of viruses is usually associated with different respiratory, hepatic, intestinal, or neurological diseases (Weiss and Navas-Martin 2005). The famous members of this family are severe acute respiratory syndrome (SARS-CoV), discovered in China in 2002, and Middle East Respiratory Syndrome (MERS) coronavirus (MERS-CoV), identified in the Middle East region in 2012, which are capable of initiating a pandemic state of pneumonia with high mortality rate up to 35% in a short time (Zaki et al. 2012; Chan et al. 2015; Draz and Shafiee 2018). In the current outbreak of COVID 19, SARS-CoV-2 originates from "reservoir of bats" and anonymous intermediate hosts (Guo et al. 2020); therefore, WHO and public health guidelines should restrict the use of wild and exotic animals like bats as food to avoid the emergence or re-emergence of viral infections of zoonotic viruses. Unfortunately, the suggested standard anti-viral drugs in clinical trials, such as chloroquine and remdesivir, administrated to positive COVID-19 cases, were not effective enough, to fight against the new virus (Guo et al. 2012; Wang et al. 2015b); the search for an efficient treatment against COVID-19 pneumonia is still open.

There are limitations to develop a technical system for virus-detection, including the simple structure and nano-scale size of viruses, which can be visualized using electron microscopy (EM) with a high-magnification power of ~ 100, 000 xs (Alizadeh et al. 2017; Yin et al. 2017). However, the detection of viruses using EM is clinically unsuitable and impractical because of safety concerns, time-consumption, and high-cost. In addition, viruses are characterized by "an extraordinary genetic adaptability" that allows them to deceive both the detection system and the anti-viral inhibition mechanism, to gain resistance against anti-viral agents (Domingo 2010) and to modify their behavior. Moreover, the growing number of discovered viruses and recorded viral infections are regarded as limitations for establishing specific diagnostic tools for viruses (Zengin et al. 2017; Yin et al. 2017).

On the other hand, several anti-viral drugs were used (Table 2), either alone or in combination, to treat CoVs-related pneumonia, for example, drugs such as interferon, ribavirin, and corticosteroids were used to treat SARS or MERS- infected cases, however, these drugs were not potent enough as anti-viral agents (Zumla et al. 2016). The anti-viral therapeutic mechanism aims at targeting the initial stage of "virus entry"; therefore, the action site of the virucidal agent is extracellular. There is a dare need to develop multifunctional anti-viral agents or vaccines with low-toxicity and broad-spectrum efficacy to encounter the emergence and re-emergence of viral outbreaks, knowing that the vaccines require pre-clinical trials and about 10 months for approval and commercialization.

Concerning the search for efficient anti-viral drugs against COVID-19, a study by Wang et al. (2020a) found that both remdesivir and chloroquine showed an in vitro anti-COVID 19 activities. Remdesivir, a nucleotide analogue, has been recently recognized as a promising anti-viral drug through acting at a post-viral entry-stage and enhancing pre-mature termination (Wang et al. 2020a; Warren et al. 2016) in RNA viruses such as SARS/MERS-CoVs in pre-clinical trials (Sheahan et al. 2017). Chloroquine, the anti-malarial and autoimmune disease drug, has recently been described as a potential broad-spectrum antiviral drug (Yan et al. 2013), through elevating endosomal pH -required for virus/cell fusion- and interfering with the process of glycosylation of viral receptors (Vincent et al. 2005) and subsequently blocking viral spread and infection. Chloroquine acts at both entry and post-entry stages of the novel CoV-19 virus in Vero E6 cells (Wang et al. 2020a). The mechanism underlying the in vivo anti-COVID-19 potential of chloroquine might be attributed to its immunomodulating and anti-inflammatory activities as well as its high distribution in different body organs including lungs (Wang et al. 2020b; Savarino et al. 2003). It was demonstrated that combining of remdesivir and chloroquine effectively inhibits COVID-19 in vitro (Wang et al. 2020b). However, performing pre-mature and unplanned clinical trials in detected cases using these drugs will present a health-care risk, as research for therapeutic and prophylactic agents are still ongoing and data have not yet been made available, besides lack of evidence-based studies. Table 2 demonstrated some of the used standard anti-viral drugs during the current pandemic of COVID-19 with all the pros and cons.

COVID-19 is easily transmissible; therefore, there is a keen interest to establish rapid and direct assays for detection of the viral replication with acceptable specificity and sensitivity. Therefore, theranostics-based approach, that combines both an efficient anti-viral therapy and a specific diagnostic system, could be a promising solution in order to conquer COVID-19. Theranostics could promote the personalized "patient-specific" treatment against SARS-CoV-2; this might be ascribed to the possible detection of pathological conditions using "MicroRNAs (miRNAs) expression profiles" as circulating miRNA is detectable in body fluids and can be used as non-invasive biomarkers (Matin et al. 2016). This review presents the metal-based nanotheranostics as a potential strategy against the viral infections of COVID-19.

### Nanotechnology applications in viral infections

**Fig. 1:**
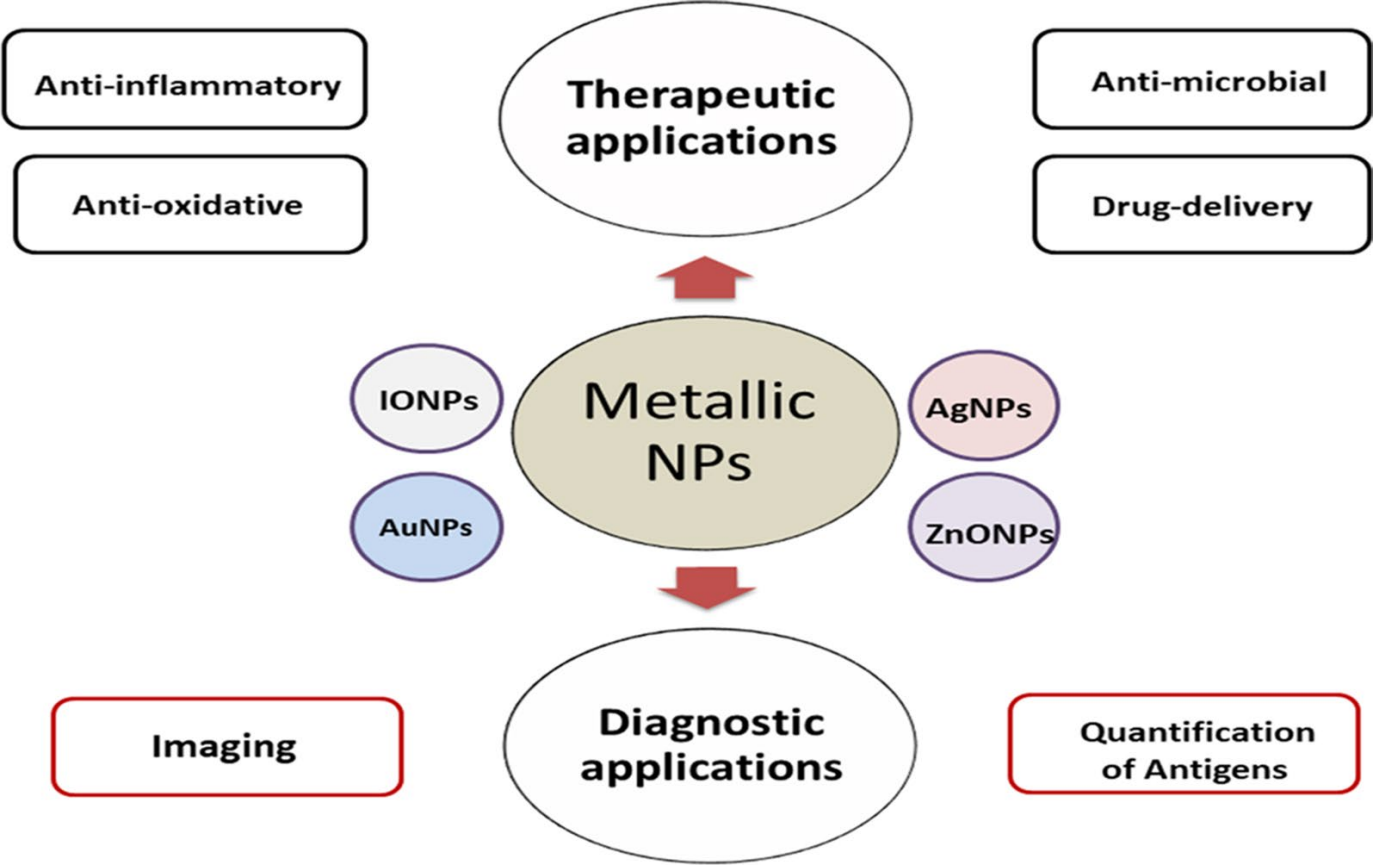
Theranostics applications of metallic nanoparticles (NPs). Metal-based NPs exhibit several therapeutic benefits including anti-microbial (e.g. anti-viral), anti-oxidative, and anti-inflammatory activities. Metallic NPs could serve as drug carriers to deliver therapeutic agents or as diagnostic tools to enable noninvasive imaging or detection of antigens

Nanotheranostics is an innovative combination of therapeutic and diagnostic functions in a single multifunctional nano-platform towards pathological conditions including cancer and neurodegenerative disorders (Kundu et al. 2019; Luo et al. 2020). For example, nanotheranostics demonstrated a great potential for the management of cancer and multiple sclerosis (MS) through enabling detection, image-guided treatment, and estimating therapeutic response (Liu 2018; Ojha and Babita 2018). Recently, theranostic NPs are regarded as innovative tools to promote targeted delivery for different active therapeutic molecules, including drugs and vaccines. Moreover, they enable visualizing and tracking both the mechanism of infection and treatment using non-invasive imaging approaches (Itani et al. 2020), as represented in Fig. 1.

Multifunctional NPs are regarded as promising theranostic agents because of their physicochemical features such as size, charge, solubility, and ease of synthesis, in addition to targeted drug delivery (TDD), biocompatibility, biodistribution, biodegradability and enhanced retention inside tissues of interest (Singh et al. 2017a). To design a promising "theranostics based nano-platform", three factors should be selected appropriately, including the therapeutic agent, the nanocarriers, the imaging agent (Madamsetty et al. 2019), biosynthesized NPs carrying therapeutic molecules and imaging agents demonstrated theranostics activities (Ovais et al. 2018; Ma et al. 2018; Vemuri et al. 2019). Therefore, certain NPs might represent both an effective anti-viral treatment and a sensitive diagnosis for SARS-CoV-2 induced COVID-19.

The unique physicochemical features and surface modifications property of metallic NPs promoted the synthesis of multifunctional NPs (Barabadi et al. 2019). For example, gold NPs (AuNPs) and magnetic NPs (MNPs) are the most commonly used nanotheranostics agents due to their outstanding features (Singh and Sahoo 2014; Liu 2018). MNPs could be used either as contrast agents (CAs) in magnetic resonance imaging (MRI) or as drug carriers (Singh and Sahoo 2014).

Currently, there are several methods for assaying the anti-viral activity of NPs, including plaque assay, the β-galactosidase assay, the confocal-imaging assay, transmission electron microscopy (TEM), Western blot assay, flow cytometry, and RT-PCR (Chen and Liang 2020). However, most of these techniques are unsuitable for clinical use or point-of-care diagnostics because of high cost, lack of specificity and sensitivity, time-consumption, and the need for well-equipped laboratory facilities and highly trained technologists (Justino et al. 2016).

Theranostics application of anti-viral NPs for the sensitive diagnosis and specific treatment of viral infections is a seeking priority in the case of the COVID-19 pandemic. Nanotheranostics will be of great potential for better management of novel emerging COVID-19 through promoting the development of NPs-based detectors or biosensors, in addition to enhancing the delivery of anti-viral agents. There are several nano-based approaches for detecting viral infections, for example, Chen et al. (2020) developing a rapid and sensitive nano-based diagnostic approach employing "lanthanide-doped polysterene NPs (LNPs)" to recognize anti-SARV-CoV-2 IgG in human serum. Ma et al. (2012) had developed a glass carbon electrodes-based immunosensor, using AuNPs and zirconia NPs, in a chitosan nanocomposite, to detect antigen (Ag) of hepatitis C virus (HCV). Moreover, the optimized field-effect transistors (FET) instruments are used to detect the nucleocaspid protein of SARS as a biomarker of the viral infection (Justino et al. 2016).

Regarding anti-viral therapeutics, metal complexes can act as nanocarriers for delivery of therapeutic agents, with improved pharmacokinetics and reduced toxicity (Sportelli et al. 2020a), for instance the 8-hydroxyquinoline-metal complexes demonstrated anti-viral potential (Phopin et al. 2016).

Targeted drug delivery (TDD) contributes to a vital advancement in the course of different pathologies, including viral infections (Singh et al. 2017b). For instance, an effective concentration of active molecules can be formed through loading of conventional anti-viral drugs or bioactive phytochemicals onto NPs (Milovanovic et al. 2017), to enable a site-specific, sustainable, and controlled treatment (Panyam and Labhasetwar 2004).

Using NPs for the management of viral infections requires the understanding of the possible interactions between the active therapeutic molecules and NPs, for example, chloroquine reduces in vitro and in vivo accumulation of synthetic NPs of different sizes and shapes (Wolfram et al. 2017). A study by Lin et al. (2017) showed that the anti-viral activity against H1N1 influenza virus of zanamivir-modified selenium NPs (Se NPs-ZNV) is higher than that of selenium NPs (SeNPs) and zanamivir alone. In addition, Li et al. (2012) demonstrated that oseltamivir surface-modified SeNPs (Se NPs-OTV) possess greater anti-viral potential and lesser cytotoxicity than that of Se NPs against H1N1 influenza virus.

Nutra-nanoceuticals, the nano-formulation of nutraceuticals, are expected to improve the delivery of active substances at specific target sites, to increase their bioavailability, to control the release of the drug, and to guarantee sustainable and effective treatment (Chellaram et al. 2014; Kakkar et al. 2016). For instance, the bioavailability of quercetin, a polyphenol with anti-inflammatory and anti-viral activities, was enhanced via using maltodextrin fast dissolving films for synthesizing "quercetin nanocrystal delivery" (Lai et al. 2015).

Moreover, several metallic NPs showed anti-viral potential, for example, silver NPs (SNPs), copper NPs (CuNPs), and gold NPs (AuNPs) (Galdiero et al. 2011). Therefore, anti-viral nano-therapeutics will present non-expensive and more accessible treatment especially in the cases of viral outbreaks of COVID-19 that require rapid intervention.

The current advances in the NPs-based delivery systems could be a promising and effective technique to deliver anti-viral agents in a controlled and sustainable release of active therapeutic molecules that target viruses, with low or minimal cytotoxicity. Coupling the anti-viral potential of metal NPs with the anti-inflammatory, anti-oxidant, and anti-viral activities of phytochemicals, such as quercetin, or drugs, such as Chloroquine, is expected to be a leaping step towards COVID-19 eradication. Future studies should involve pre-clinical and clinical investigations to test the validity of NPs-based delivery systems against COVID-19. The metallic NPs could represent potential nanotheranostic agents for viral inhibition and detection (Table 1).

### Metal nanoparticles as potential anti-viral nanotheranostic agents

#### Silver NPs (SNPs)/AgNPs

The anti-viral potential of metallic NPs was firstly described by Elechiguerra and his colleagues (Elechiguerra et al. 2005), they studied a size-dependent interaction of SNPs (1–10 nm) with Human immunodeficiency virus 1 (HIV-1) and showed that the favorable binding of SNPs to the gp-120 glycoprotein knobs, demonstrating the in vitro anti-viral potential. Another study by Lara et al. (2010) investigated the in vitro anti-viral potential of SNPs and suggested that these NPs act as virucidal agents through exerting anti-HIV-1 action at the initial phase of viral replication and inhibiting the post-entry phase. The anti-viral action of SNPs might be ascribed to their ability to block viral proteins or to suppress the process of the reverse transcription (Galdiero et al. 2011). Combination of SNPs (AgNPs) with nutraceuticals is a promising source of novel anti-viral agents, because of the "multi-targeting and multi-directional" mode of action (Orłowski et al. 2018). The use of phytochemicals such as tannic acid (a polyphenol with anti-viral action) in combination with SNPs showed a high virucidal action; TA-SNPs were capable of treating the genital herpes infection through boosting a virus-specific immunological response and limiting the viral spread and recurrence (Orłowski et al. 2018). TA modified-silver/copper NPs (TA-SNPs and TA-CuNPs) showed both in vitro and in vivo anti-herpes simplex type 2 (HSV-2), an enveloped virus, through directly blocking virus penetration into cells and suppressing inflammation (Krzyzowska et al. 2017). Moreover, TA-SNPs-hydrogels demonstrated anti-HSV activity against HSV-1 and HSV-2, through directly inhibiting viral entry, infection, and spread (Szymańska et al. 2018). Previously, Lu et al. (2008) showed that SNPs are capable of inhibiting the replication of the Hepatitis B Virus (HBV) through direct interaction with viral particles. The SNPs-based virucidal mechanism varies virus to virus; SNPs are capable of inhibiting viral replication as in dsRNA viruses or suppressing viral entry into host cells as in HIV-1 (Kerry et al. 2019).

AgNPs-fixed on graphene oxide (GO) sheets (GO-AgNPs) demonstrated anti-viral activity against enveloped and non-enveloped viruses, with low cytotoxicity (Chen et al. 2016). GO and GO-SNPs could be identified as anti-viral and virucidal agents against the enveloped feline coronavirus (FCoV), through forming interactions between "negatively charged" GO-SNPs and "positively charged" lipid membranes of the virus and increasing the adsorption of more lipid membranes, leading to the rupture of viruses (Barabadi et al. 2019). SNPs are ideal candidates for developing nanotherapeutics against several viral infections (Xiang et al. 2011; Mori et al. 2013; Gaikwad et al. 2013; Chen et al. 2013; Huy et al. 2017) (Table 1).

#### Gold NPs (Au NPs)

Drug-loaded gold NPs (AuNPs) aimed at intracellular inhibition mechanisms of viral activities. AuNPs should interact with virus particles to demonstrate better anti-viral actions, through inhibiting viral entry (Yaqoob et al. 2020). For instance, using AuNPs for delivery of antiviral peptide "FluPep" improved its solubility and resulted in a synergistic anti-viral potential against influenza virus (Zaid et al. 2018). Moreover, conjugation of AuNPs with peptide triazole demonstrated anti-viral activity against HIV-1 (Emileh et al. 2013). Furthermore, porous gold nanoparticles (Po-AuNPs) could be used for detection of Influenza viruses, besides their anti-viral activity (Kim et al. 2020).

Some biocompatible polymers-stabilized AuNPs demonstrated virucidal potential against HIV-1 and influenza virus (subtypes: H1N1, H3N2, H5N1) (Kerry et al. 2019). Papp et al. (2010) investigated the anti-viral action of sialic acid (SA)-modified AuNPs, 2-nm and 14-nm sizes, to inhibit the attachment of influenza virus H1N1 to the cellular membrane. The 14-nm AuNPs demonstrated a higher anti-viral potential, proving that this activity is size-dependent and relies on the spatial distribution of the interacting molecules.

Moreover, AuNPs were regarded as ideal nanodiagnostic tools for viral infections; AuNPs are chemically stable, feasible, biocompatible, simply synthesized, with a large surface area-to-volume, and easy surface-modification (Baptista et al. 2008; Lin et al. 2009). It was found that cationic AuNPs are capable of detecting H1N1 and H3N2 (Ahmed et al. 2016). In addition, AuNPs could be functionalized to act as highly sensitive and specific labeling/detective markers due to their ability to form active and stable bioconjugates, with biomolecules such as DNA, which can be easily detected (Baptista et al. 2008). For example, unmodified AuNPs were used to enable colorimetric detection of DNA sequences; AuNPs are capable of adsorbing single-stranded DNA over double-stranded DNA, this could be regarded as an accurate and sensitive "fluorescence-based assay" (Li and Rothberg 2004).

AuNPs were used to detect SARS to establish a sensitive and fast molecular detection through an assay for detection of "pp1ab" gene colorimetrically and detection of "nucleocapsid protein" gene electrochemically; "SARS-specific DNA-captivating probes" are fixed on AuNPs-structured electrodes; then, they are allowed to hybridize with the biotinylated targets (Shawky et al. 2010). AuNPs-based colorimetric assay for SARS detection is easy, simple, highly sensitive, and time- saving technique that allows the early detection of SARS infection (Shawky et al. 2010). Moreover, AuNPs can be employed for the high sensitive and selective detection of "synthetic HCV sequences" due to their ability to quench fluorescent dyes (Griffin et al. 2009a, b). Kim et al. (2019) developed a simple and rapid colorimetric method that depends on AuNPs that uses disulfide-induced self-assembly and consists of two thiol-modified probes for detecting genomic regions (30 bp) of infectious MERS-CoV, within 10 min. This non-expensive detection assay will be of great value in regions of a pandemic. Therefore, developing AuNPs-based assays will provide rapid, sensitive, non-expensive, direct, and simple diagnostic tools of unamplified nucleic acid extracted from human specimens. These AuNPs-based assays could act as diagnostic tools for the detection of viral replication rather than traditional detection systems such as RT-PCR.

**Fig. 2:**
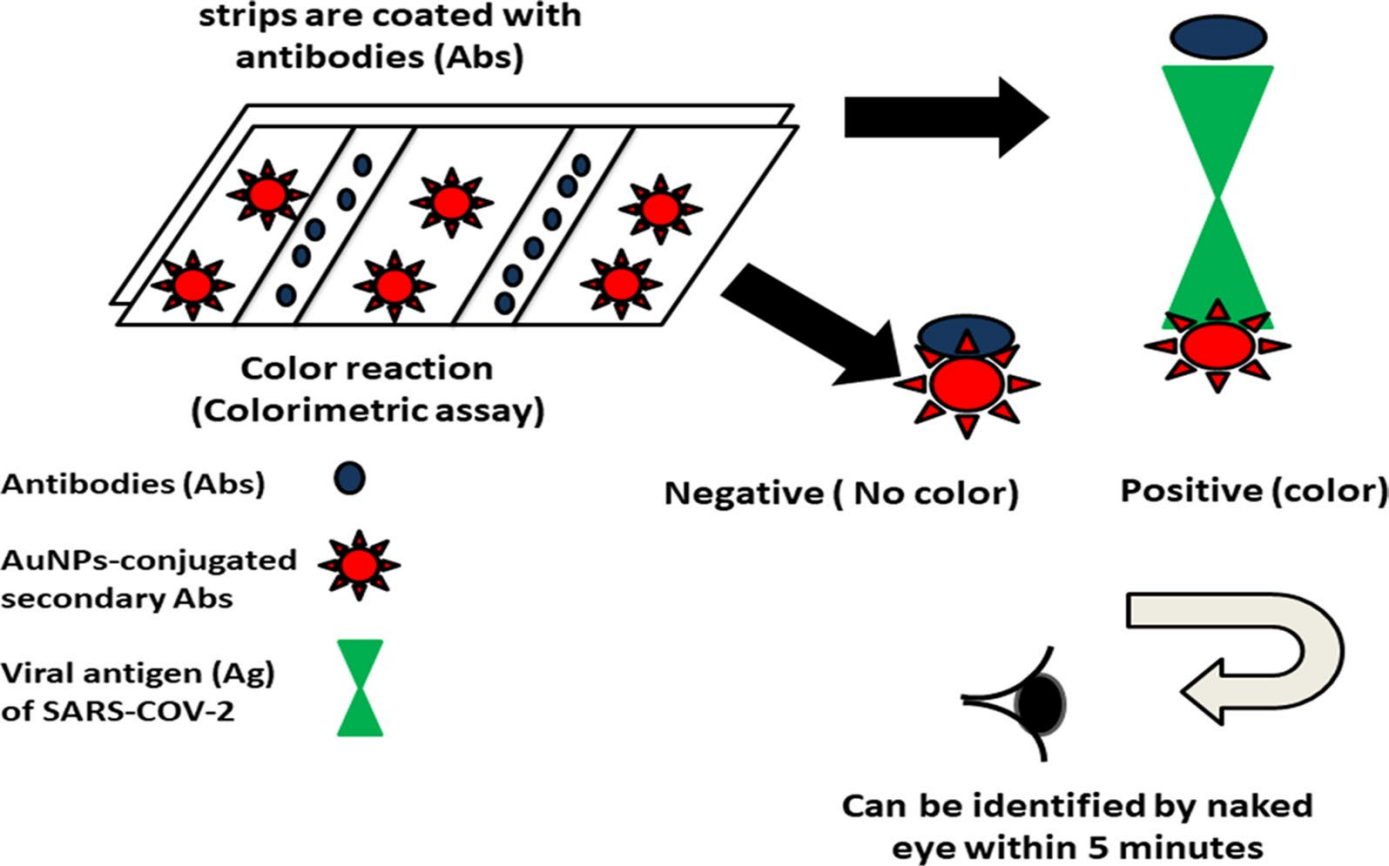
Schematic illustration represents the AuNP-based strips for the colorimetric detection of severe acute respiratory syndrome (SARS). Strips are coated with antibodies (Abs) that are capable of binding to viral antigen (Ag) of SARS-COV-2, that subsequently aggregate with AuNPs-conjugated secondry Abs. This aggregation results in color reaction, indicating the positivity of the tested sample

Recently, companies including the World Nano Foundation (WNF) and MIT-spin out startup are developing AuNPs-based strips and a rapid "IgM/IgG antibody assay kit" for COVID-19. The main idea is based on using AuNPs-based strips. The strips are covered with antibodies (Abs) that interact with the viral particles of SARS-COV-2 (viral antigen), while the second Abs are conjugated with AuNPs. The presence of viral antigen in sample (e.g. blood and urine) will result in color reaction (Chakravarty and Vora 2020; Udugama et al. 2020), as illustrated in Fig. 2.

AuNPs-based strips are commercial lateral flow assays that showed a clinical sensitivity (57 and 81%), specificity (100 and 100%), and accuracy (69 and 86%), for IgM and IgG, respectively (Udugama et al. 2020).

AuNPs are widely used in immunotherapy applications, as these NPs are able to inhibit antibody production against the platform material, to stimulate several immune cells, and to enhance the generation of pro‐inflammatory cytokines (Dykman and Khlebtsov 2017; Sekimukai et al. 2019). However, more research is required to test the efficiency of the "AuNPs‐adjuvanted vaccine" against COVID-19 infection (Sekimukai et al. 2019).

In addition, AuNPs, in the presence of H5N1 particles, aggregate and change color from bright red to purple spectroscopically (Sajjanar et al. 2015). Moreover, Chiodo et al. (2014) developed a highly active anti-retroviral therapy (HAART) that used carbohydrate-coated AuNPs loaded with anti-HIV abacavir and lamivudine, in a pH-mediated release model, "HAART for HIV" acts through inhibiting the viral replication. AuNPs displayed potential nanotheranostics agents for the management of viral infections (Gopinath et al. 2013; Paul et al. 2014; Tao et al. 2014; Liu et al. 2015; Askaravi et al. 2017; Lysenko et al. 2018; Wang et al. 2018) (Table 1).

#### Zinc oxide NPs (ZnO NPs)

Zinc oxide NPs (ZnO NPs) are characterized by being non-cytotoxic, biocompatible, and accessible (Sirelkhatim et al. 2015). However, there are few studies about the anti-viral actions of ZnO NPs; most studies have focused on their microbicidal (anti-bacterial) potential. A study by Ghaffari et al. (2019) investigated the anti-viral activity against H1N1 and demonstrated that polyethylene glycol (PEG)-coated ZnO-NPs (PEG-ZnO NPs) have a higher anti-viral action and lower cytotoxicity than non-pegylated "bare" ZnO NPs. This potential might be attributed to the "pegylation of NPs: coating of the surface of NPs with polymeric polyethylene glycol" that promotes the anti-viral activity against H1N1 influenza and the anti-oxidant potential, leading to viral inhibition coupled with decreased toxicity. ZnO NPs might act as effective antiviral nanomaterials (Antoine et al. 2012; Ghaffari et al. 2019) (Table 1).

#### Copper NPs (CuNPs)

Copper NPs on a polymer structure demonstrated a high anti-microbial potential via disrupting the cellular membranes of microorganisms adsorbed on the surface through promoting the interaction between microbial biomolecules and copper ions (Palza 2015), demonstrating the biocidal potential of copper nanocomposites in polymers (Zuniga and Cortes 2020). The controlled and sustainable release of bioactive copper ions and the associated oxidative stress are responsible for the anti-viral potential of copper NPs against coronaviruses (Sportelli et al. 2020a; Van Doremalen et al. 2020). Moreover, it was found that "quaternary ammonium shell enclosing a copper" could act as synergistic nanovirucidal agents (Sportelli et al. 2020b).

#### Cerium dioxide NPs (Nano-ceria)

Cerium dioxide (CeO_2_) is one of the promising nanomaterials that could be incorporated into the synthesis of nanovaccines (Zholobak et al. 2011); attaching antigens with nanocarriers could enhance the effectiveness of vaccines and increase antigen uptake by immune cells (Wibowo et al. 2014), regarding that nano-ceria demonstrated anti-oxidative potential coupled with negligible toxicity (Zholobak et al. 2016). Moreover, the use of nano-ceria can improve the biological activity of interferon (IFN)-one of the components of the influenza vaccines- (Zholobak et al. 2016). Therefore, more research is required to investigate if ceria-modified vaccines could be effective against SARS-COV 2 by providing a long-lasting immune response.

#### Silica NPs (SiNPs)

Si NPs could be considered as one of the promising nanotheranostics for controlling viral infections. SiNPs demonstrated anti-viral activity against some viruses such as Hepatitis B virus, Human Papilloma Virus, Human immunodeficiency virus, and recombinant viruses (Kerry et al. 2019). SiNPs act as anti-viral agents through blocking the virus entry or through immunization of the host (Guo et al. 2012; Hanchuk et al. 2016). The use of functionalized SiNPs is an innovative strategy for viral infection.

Moreover, SiNPs could be used for the detection of viral infections through "Viral protein-based fluorescent detection" or " nucleic acid hybridization" (Chunduri et al. 2017). Fluorescent SiNPs were functionalized with lanthanide chelates for developing "highly sensitive time resolved immuno-fluorometric assays" (Xu and Li 2007). SiNPs-enhanced microcantilever sensor was used to enable detection of viral DNA of HBV (Lee et al. 2010). In addition, the non-expensive and rapid fluorescents dye tagged-SiNPs is used to detect hepatitis in serum of infected patients (Bae et al. 2012). Surfactant-modified nanoclay, with nano-silicate platelets, demonstrated anti-viral activity against influenza A virus (Liang et al. 2014). SiNPs is capable of acting as viral binding/entry inhibitors against Herpes simplex virus (HSV) infections caused by HSV-1 and HSV-2 (Lee et al. 2016).

#### Other NPs

The anti-viral activity of different metallic NPs, such as iron oxide NPs (IONPs), Graphene oxide (GO), and selenium NPs (SeNPs) and zanamivir -modified selenium NPs (SeNPs-ZNV), against respiratory viral infections was evaluated in several studies (Lin et al. 2017; Yang et al. 2017; Kumar et al. 2019). The suggested mechanism is the interruption of the life cycle of the virus via suppressing of hemagglutinin and neuraminidase activities (Ghaffari et al. 2019).

Superparamagnetic iron oxide NPs (SPIONPs) function both as magnetic anchors, toward desired molecules, and as contrast agents for MRI (Yadavalli and Shukla 2017). Also, lipids-coated SPIONPs are capable of delivering the anti-viral agents to targets of interest (Al-Jamal and Kostarelos 2007). Several studies demonstrated the nanodiagnostic potential of IONPs (Chou et al. 2011; Shelby et al. 2017) (Table 1).

Titanium nanoparticles (TiNPs) demonstrated anti-viral activity limited to influenza virus (H3N2), the direct interaction of TiNPs with virus particles result in inhibition of the viral activity (Mazurkova et al. 2010). In the current pandemic state, the streets in Milan (Italy) were disinfected using a solution containing titanium dioxide and silver ions (Nanotech Surface Coronavirus 2020). TiNPs demonstrated anti-viral potential against the H9N2 avian influenza virus (Jiang et al. 2009). Moreover, DNA tagged-TiNPs inhibited viral replication of H5N1 and H1N1 viruses (Levina et al. 2015).

Whilst several approaches are being developed to decrease the infection rate of COVID-19, there is an urgent need to treat the increased detected cases that developed pneumonia and to detect asymptomatic cases. In this review, we demonstrated the use of metallic nanoparticles as theranostic agents to control the COVID-19 pandemic through enabling treatment and detections of viral infections.

### The mechanism underlying the anti-viral action of metal NPs

Metallic NPs could act as virucidal agents that are capable of blocking viral spread, interacting with viruses, and suppressing free virions (complete virus particles) (Zhou et al. 2020). This anti-viral potential should be supported with the immunological potential for provoking a virus-specific immunity, with no or minimal cytotoxicity. This would aid in accelerating the development of vaccines and therapies to accommodate the outbreak of the coronavirus pandemic state. This review supports the potential application of metallic NPs as anti-viral theranostics against SARS-COV-2, through presenting evidence-based data.

Functionalized metallic NPs act as antiviral agents by blocking the virus attachments and entry into the host cells (Cojocaru et al. 2020). For example, SNPs demonstrated specific interactions with pathogenic viruses and bacteria (Verma and Maheshwari 2019). The microbicidal potential of SNPs declines as the size increases, spherical SNPs (< 10 nm) possess a greater antiviral action against HIV-1 (Elechiguerra et al. 2005; Xiang et al. 2011), smaller SNPs demonstrated a greater anti-viral potential, especially when < 100 μg of Ag NPs was added to 1 mg of chitosan activity (Mori et al. 2013). Moreover, SNPs demonstrated anti-herpes simplex virus (HSV) types 1 and 2 and anti-human para-influenza virus type 3 (Gaikwad et al. 2013). In addition, "small-sized Tannic Acid-SNPs" helped to better internalize HSV-2 antigens (Orłowski et al. 2018). For instance, it was found that complex anti-viral NPs demonstrated minimal or low cytotoxicity in vivo (Lysenko et al. 2018). Due to their small size, SeNPs are often used for loading of drugs or siRNA (Lin et al. 2017). The higher antiviral potential of IONPs was at lower dose; this might be attributed to the small size that enable direct interaction of NPs with protein knobs of Influenza virus, in addition to the regular spatial placement of bound NPs (Kumar et al. 2019). In addition, ZnO-NPs of smaller sizes demonstrated a high anti-bacterial potential (Zhang et al. 2007). We could assume that the microbicidal potential of NPs is a size-dependent activity, the smaller the size, the higher the potential. This might be attributed to the "spatial restriction of binding" between NPs and vial particles (Elechiguerra et al. 2005).

The anti-viral potential of NPs could be attributed to their adsorption onto the viral surfaces and inducing subsequent local transformations such as "agglutination of glycoproteins", and thus blocking virus penetration and entry into host cells (Rafiei et al. 2015; Lysenko et al. 2018). In addition, local-field action against the viral receptors is regarded as the critical mechanism of NPs-mediated antiviral action; it was assumed that there could be several configurations in the "NP-virus" interaction, which results in mitigating the potential of one nanoparticle and thus disappearing of domains of the enhanced local field (Lysenko et al. 2018). Therefore establishing a stable "NP-virus" interaction would be a vital stage that explains the anti-viral actions of NPs. For example, Cagno et al. (2018) demonstrated that engineering the linkers of anti-viral NPs could transfer the inhibitory action from "viru-static" to "viru-cidal" and result in the death of viruses.

The main mechanism underlying the anti-viral potential of NPs is the direct interaction of NPs with the viral surface proteins and the viral genome (Galdiero et al. 2011). The size of NPs is one of the main factors that affect the anti-viral action of NPs; for instance small-sized NPs showed more anti-viral potential than larger ones through blocking of viral replication (Khandelwal et al. 2014).

Another proposed mechanism is the inhibition of generic expression and production of viral constituents, for instance, green-synthesized SNPs suppressed the expression and subsequent production of envelope (DEN-2) protein of Dengue Virus in vitro (Murugan et al. 2015).

In addition, polymeric NPs are ideal candidates for the treatment of several pulmonary pathological conditions such as lung cancer and tuberculosis; therefore, these NPs might be efficient against COVID-19 associated pneumonia. Synthetic and polymeric NPs, using poly lactic-co-glycolic acid (PLGA), are biocompatible, biodegradable, and surface adaptable nanocarriers (Li et al. 2012). Polymeric NPs of two poorly soluble anti-viral drugs were prepared according to Mazumder et al. (2017), who found that polysaccharides-drug combinations improved the solubility and ameliorated the release of drugs loaded within NPs.

NPs can promote targeted drug delivery (TDD) by applying surface modification (encapsulation or coating) against immune response, enhancing cellular uptake, provoking apoptosis of cancer cells, and blocking pathways for lung cancer initiation and progression (Bonner 2016), assuming that these mechanisms could work with pneumonia-associated COVID-19 through enhancing the selective toxicity of NPs for virally infected cells. Therefore, understanding the underlying mechanisms of NPs and the physicochemical features of NPs could facilitate directing the NPs-associated oxidative stress or apoptosis towards affected cells with pneumonia or cancer and induce their programmed death, rather than affecting normal (healthy) cells (Barcińska et al. 2018).

Viruses are “nanoscale objects” that could be considered as natural nanomaterials; thereby NPs-based approaches are of great importance in vaccine technologies (Shin et al. 2020). “Nano-scale delivery systems” are supposed to play an essential role in the development of these nano-based prophylactic and therapeutic strategies (Florindo et al. 2020). Therefore, the interaction of NPs with virus presents a great potential in both nano-diagnostic and nano-therapeutic approaches (Talebian and Conde 2020). For example, incorporation of NPs in the vaccine formulations "nanovaccines" can lead to a sustained and controlled release of antigens or adjuvants, as well as, prolonged shelf-life (Zhao et al. 2014; Singh et al. 2017a). The use of nanovaccines might achieve three main goals: (1) improved Ag stability by protecting them from degradation by proteolytic enzymes (2) enhanced immunogenicity, to produce ABs to deactivate viruses and (3) targeted delivery using NPs to promote Ag uptake and processing by antigen-presenting cells (APCs) (Talebian and Conde 2020). Therefore; nanovaccines can further improve the therapeutic potential of vaccines to fight the current pandemic of COVID-19 (Talebian and Conde 2020). In addition, NPs-based approaches represent an important tool to improve the stability and ameliorate the pharmacokinetics of therapeutic Abs (Florindo et al. 2020).

NPs are ideal for antigen delivery “as adjuvants” or through mimicking the morphological characteristics of viruses (Shin et al. 2020). NPs are modified in formulations of nanovaccines and nanodrugs to act as immunomodulators, through promoting antigen (Ag) presentation and/or as immuno-stimulatory adjuvants. The application of nanotherapeutic approaches for the management of viral infections enhances the potential of available nanovaccines and nanodrugs (Singh et al. 2017b), for instance RNA vaccines carried by lipid NPs have already reached Phase II and III clinical trials (Shin et al. 2020); thus this nano-based vaccine development will be of great benefit in states of pandemic.

### Nanotoxicity

The major limitations of nanotherapeutics are the long-term cytotoxicity and heterogeneity of the preparation (Ojha and Babita 2018). Understanding the potential cytotoxicity, biocompatibility, biodistribution, identification of "NP-virus" interaction and "NP-cell" biological interaction is mandatory to develop efficient nanotherapeutics approaches (Singh et al. 2017b; Ojha and Babita 2018).

Moreover, other issues such as solubility, retention, accumulation in tissues, and the release of metal ions of dissolved NPs may result in undesirable outputs such as oxidative stress, programmed cell death, mutagenesis, and cytotoxicity (Kreyling et al. 2010; Li et al. 2012; Mazumder et al. 2017). Some experiments have shown that SNPs induced cytotoxicity and genotoxicity (AshaRani et al. 2009; Kawata et al. 2009).

Nanotoxicity involves factors such as deposition, circulation time, and clearance time of NPs that may result in severe side effects that affect many organs including hepatotoxicity, renal failure, neurotoxicity, and pulmonary fibrosis (Singh et al. 2017b). Clinically, the use of "gold nanorods" in patients infected with hepatitis could result in hepatotoxicity (Bartneck et al. 2012).

Oxidative stress is strongly implicated in nanotoxicity, as well as in structural and cellular alterations related to cytotoxicity, apoptosis, oxidative DNA damage, DNA strand breaks, micronucleus formation, uncontrolled cellular signaling, damage to cellular membranes, and carcinogenesis (Shukla et al. 2011; Mei et al. 2012; Zhu et al. 2013; Pinzaru et al. 2018). NPs have unique physicochemical features (e.g. size, surface area, and shape) that correlate with their oxidative activity (Fu et al. 2014; Abdal Dayem et al. 2017).

**Fig. 3:**
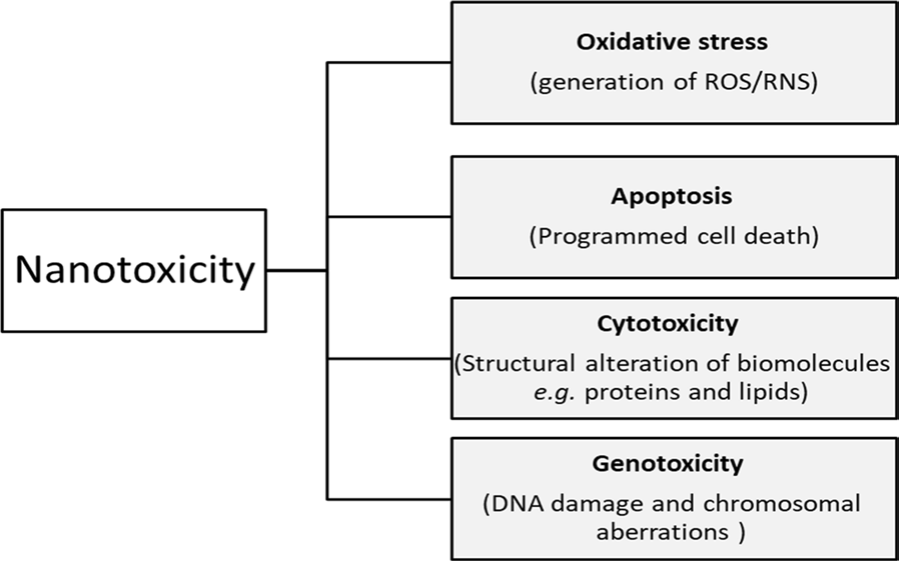
Schematic illustration demonstrates the consequences of nanotoxicity. NPs-correlated toxicity could result in oxidative stress caused by ROS generation, apoptosis, cytotoxicity, and genotoxicity. The anti-viral potential of NPs might be ascribed to these nanotoxicity-associated mechanisms, through enhancing mitochondrial-apoptotic pathways. In case of non-selective toxicity, these mechanism might cause damage to the mammalian cells

Metal-based NPs are capable of inducing oxidative damage to DNA and chromosomal aberrations (Xie et al. 2011), through interaction of generated free radicals (HO·) with DNA and the production of 8-hydroxyl-20-deoxyguanosine (8-OHdG), which is a marker of oxidative stress and carcinogenesis (Valavanidis et al. 2009). In addition, oxidative stress results in oxidation of polyunsaturated fatty acids (Zhu et al. 2013). Lipid peroxidation is strongly co-related with metal NP-induced genotoxicity (Shukla et al. 2011). Besides, metal NPs are capable of generation an oxido-inflammatory state that is characterized by activation of inflammatory mediators e.g. cytokines (Shvedova et al. 2012). NPs are capable of interfering with the genetic expression of both oxidative stress-related genes and antioxidant genes (Yu et al. 2020). Iron and copper, as transition metals, can result in the oxidative stress through "one-electron oxidation–reduction reactions" and the release of metal ions (Yin et al. 2012). Figure 3 depicts the different mechanism of action of NPs-associated nanotoxicity.

**Fig. 4:**
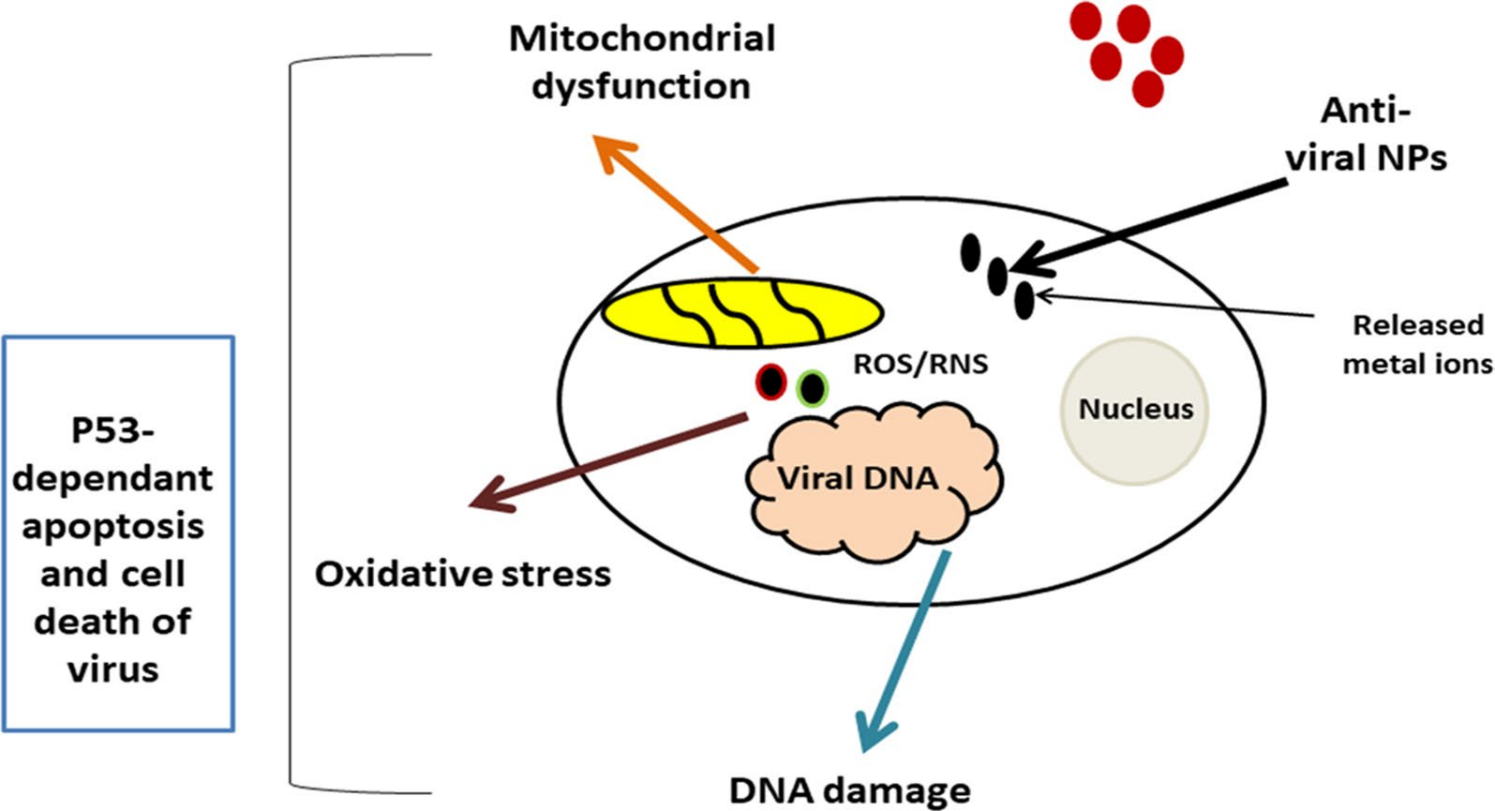
Schematic illustration demonstrates the anti-viral action of metallic NPs. The anti-viral activity of metallic NPS is largely due to the induction of oxidative stress and generation of reactive oxygen species (ROS), which finally leads to mitochondrial dysfunction, which in turn results in DNA damage via a ROS-mediated mitochondrial intrinsic p53-dependent apoptotic pathway or via the autophagy-signaling pathway

This NPs-induced oxidative stress could be used to boost the anti-microbial activities (antibacterial and antiviral) activities of NPs (Zhu et al. 2013), keeping in mind the concept pf selective toxicity that target the microbial cells. The anti-microbial action of Ag Nps, ZnO NPs, and IONPs might be attributed to the upregulation of ROS and RNS generation that leads finally to the death of microorganism (Zhu et al. 2013). This mechanism was illustrated in Fig. 4. Thereby, more research is required to investigate the biological interactions of NPs with biomolecules such as DNA, mitochondria, and cell membrane and to evaluate the in vivo safety or toxicity concerns (Elbialy et al. 2019).

This emphasizes the importance of understanding the NPs-associated ROS/RNS generation to help in the modification of physicochemical features of NPs, in order to direct oxidative stress towards specific targets e.g. viruses. For instance, the use of Ag-NPs as anti-tumor agents is ascribed to the generation of oxidative stress in cancer cells that leads to the death of tumor cells (Barcińska et al. 2018). The mitochondria determine the cellular fate of NPs-treated cells; through intracellular signaling cascades which initiate mitochondria-mediated "controlled" apoptosis, or start to fix the NPs-induced damage (Wiesmann et al. 2020). It was suggested that NPs (e.g. ZnO NPs) could selectively induce apoptosis of cancer or viral-infected cells (in this review) via a ROS-mediated mitochondrial intrinsic apoptotic pathway and enhancing p53-dependent programmed cell death or via the autophagy-signaling pathway (Jiang et al. 2018); this would be a revolutionary application of metal-based NPs. The cellular uptake and the colloidal stability of "coated NPs" are among the factors that control the toxicological features (either genotoxic or cytotoxic) of NPs against living cells (Docea et al. 2020). Therefore, investigation into the cytotoxic potentials of NPs is mandatory for enabling the safe and commercial application of NPs, to avoid inflammation, oxidative stress, genotoxicity, and apoptosis of normal cells (Abdal Dayem et al. 2017).

Several solutions were adopted to limit the nanotoxicity such as the surface modification (treatment). The surface modification is one of the mechanisms used to reduce the cytotoxicity and to enhance the biocompatibility of NPs, through using capping agents such as poly(lactic-co-glycolic acid) (PLGA), PLGA-capped NPs demonstrated lower toxicity; this could lead to optimize and control drug release, facilitate immune response, and enhance specificity to target tissues (Romero et al. 2010).

### Morphological factors of metallic nanoparticles

Beside chemical composition, also size and shape of nanomaterial are fundamental in biological activity. The biological consequences of metallic NPs depend on chemical composition, size, shape, crystallinity, geometries, surface chemistry, and aggregation. These physicochemical factors influence the biological interactions including cellular uptake, absorption through biological membranes, translocation to the target site, and the tendency to cause toxicity (Sánchez-López et al. 2020). Therefore, modulating these parameters will enable designing the required metallic NPs (Raza et al. 2016) to improve their interaction with biomolecules and facilitates their physical transfer within in vivo systems (Rasmussen et al. 2010). The nano-scale size confers larger surface-areas to NPs that demonstrate size-related physicochemical properties, as compared to bulk nanomaterials (Sirelkhatim et al. 2015).

Particle size and shape are the most important factors of nano-systems that determine the in vivo distribution, efficiency, toxicity, and destiny (Lara et al. 2011), for instance, the small size and large surface area of NPs might result in aggregation and could lead to nanotoxicity as mentioned above. SNPs of size 3 nm demonstrated a higher toxicity than that of 25 nm (Yen et al. 2009). The antiviral potential of NPs is controlled by several factors including size, shape, and capping agents (Chen et al. 2016). The small size of NPs allows them to enter into the living cells, while the coating material of nanomaterials protects the anti-viral agents from degradation (Nikaeen et al. 2020).

Besides, the shape of NPs is a key element that affects the microbicidal efficiency and modulates the cytotoxicity through affecting the mechanism of cellular uptake (Akter et al. 2018). For example, truncated triangular-shaped NPs demonstrated higher anti-microbial activity than that of rod- and spherical-shaped particles (Pal et al. 2007). This shape-dependent activity might be ascribed to "large surface to volume ratio of spherical shapes", this enables the maximum reactivity that ensures the effective interaction between NPs and viral particles (Raza et al. 2016; Albanese et al. 2012).

In addition, surface modifications of NPs influences their microbicidal efficiency (Akter et al. 2014); moreover, the larger available surface of NPs allows the surface functionalization through crosslinking with several legends thus confers several possibilities (Burdușel et al. 2018). In addition, capping material is an important factor that should be considered, for example, unsuitable capping material could render SNPs ineffective through decreasing the release of free Ag ions (Lara et al. 2010). Accordingly, surface PEGylation might enhance the antiviral activity and reduce the cytotoxicity of NPs, for instance, PEGylated ZnO-NPs demonstrated higher antiviral potential against H1N1 influenza virus, as compared to naked ZnO-NPs (Ghaffari et al. 2019). Pharmacologically, the surface coating material of NPs can be modified to control their stability, solubility, and interaction and to produce NPs of distinct physical features. In addition, the surface of NPs can be cross-linked with functional groups for several purposes, such as conjugating or tracking molecules (Her et al. 2017).

Due to their unique size-dependent physicochemical properties (e.g. high surface to volume ratio) and their "synthetic versatility", the use of NPs in nano-based systems is highly increased in several biomedical applications. Actually, electrochemical methodologies are preferred than chemical methods to synthesize noble and pure "size-selective" and "shape-controlled" NPs with required surface properties (Albanese et al. 2012). The current advancements in chemical methodologies enable the modulation of the size and shape of metallic NPs (Karami and Fakoori 2011). In the nano-system, there is a complex interaction between size, shape, and surface charge; a puzzle that should be solved (Wang et al. 2017).

The characterization of synthesized NPs is essential to generate the required NPs with unique physicochemical properties. Characterization of synthesized NPs is essential to determine their physicochemical properties to evaluate their behavior, bio-distribution, and efficacy (Zhang et al. 2016). There are several analytical techniques for characterizing NPs and determining their size and size distribution, measuring their zeta potential and estimating their surface morphology, such as atomic force microscopy (AFM), X-ray Photoelectron Spectroscopy (XPS), field emission scanning electron microscopy (FESEM), Localized Surface Plasmon Resonance (LSPR), Fourier Transform Infrared (FTIR) Spectroscopy, electrophoresis laser Doppler, and energy-dispersive X-ray (EDX) (Zhang et al. 2016). Besides, "UV–Vis Spectroscopy" is used to observe the stability of synthesized NPs, while "X-ray diffraction (XRD) is used to analyze the analysis of crystal structures, "Dynamic Light Scattering (DLS)" is used to measure narrow particle size distributions (2–500 nm), and surface-imaging tools such as Scanning Electron Microscopy (SEM) and Transmission Electron Microscopy (TEM) (Zhang et al. 2016). More of these techniques are listed in (Table 1) that demonstrated the metallic nanotheranostics for viral inhibition and diagnosis.

Finally, modulating nanomaterials with different sizes and shapes present several tools for application as nanotherapeutics or nanodiagnostics against viral infections; for example, functionalized size facilitates in vivo drug delivery across biological barriers, while surface modification facilitates cellular uptake and enhances biostability. All these factors contribute to enhanced antiviral activity through controlled drug delivery and sustainable treatment in addition to decrease drug resistance, improved pharmacokinetic and/or pharmacodynamics, and enable the development of personalized therapy (Cojocaru et al. 2020). The anti-viral potential of metal NPs holds a prominent and possible solution to the current pandemic of SARS-COV-2; NPs are capable of interacting with virus particles, either naked or in vivo, in a characteristic spatial arrangement (Galdiero et al. 2011).

### Feasibility, data gaps, and concerns of metallic nanotheranostics against COVID-19

To deal with data gaps regarding this issue; we cited relevant references (Table 1) to fill the "data gap" where the nanomaterials could be utilized as a potent antiviral therapeutic agent or as a diagnostic tool. This process of "gap-filling" was a valuable tool to assess the feasibility of metallic NPs as nanotheranostic agents against COVID-19, through providing evidence-based data, which can guide the future research works for establishing successful and effective nanotheranostic agents against SARS-CoV-2 infection.

In addition, the existing gap between "material research" and "clinical application" could be filled by employing nanomedicine (Rezvantalab et al. 2018). The outcomes of the research, either in the material science or in the pre-clinical experimentation, should be translated into safe clinical applications (Sportelli et al. 2020a). The process of "synthesis and functionalizing NPs" is feasible, effective, and reproducible; this feasibility of synthesized NPs represents a significant advantage, which enables us to improve the diagnosis and treatment of several viral infections. Nanotheranostics can fill the gap regarding the treatment and diagnosis of viral infections. Moreover, nanotheranostics could provide the biomedical field with different feasible applications regarding the current pandemic of COVID-19. Nano-based therapeutics should provide a safe and viable treatment and overcome the limitations of current therapeutic approaches, which demonstrated defects in responding to emerging pathogen outbreaks (Zhang et al. 2016). Further research work is required to assess the clinical feasibility of synthesized NPs and develop NPs that better fit the nano-based clinical applications, regarding the biocompatibility and safety concerns.

The present study addressed some limitations and concerns regarding the use of NPs for treatment and diagnosis of COVID-19 viral infections. For example, the management of viral infections (treatment or detection) is expensive, ineffective, inaccessible, and unaffordable to some countries (Cojocaru et al. 2020). The nature of the virus hinders the development of an effective management strategy, due to the unique behavior and specific viral metabolic functions (Cojocaru et al. 2020), if SARS-COV-2 has a genetic shift, as the influenza virus, this could be a limiting step to control the viral spread (Gansukh et al. 2016).

The commercialization of functionalized NPs is still limited due to concerns related to nanotoxicity (Sportelli et al. 2020a); NPs might result in undesired side effects such as nanotoxicity, inflammation, genotoxicity, oxidative injury, and finally affecting the respiratory system and resulting in pulmonary fibrosis (Gansukh et al. 2016). Besides, there are other limitations regarding NPs, such as restricted carrying capacity, the high cost, and manufacturing issues (Sivasankarapillai et al. 2020). Finally, the interaction of metallic NPs with SARS-COV-2 is a new unexplored field. Therefore, the effective and feasible management of viral infections requires boosting the safety concerns of nano-based approaches, through involving a routine "safety evaluation" assessment during the functionalizing of NPs (Sivasankarapillai et al. 2020). Moreover, an interdisciplinary platform is mandatory to facilitate clinical translation of nanotheranostics-based strategy to fight viral infections.

## Conclusion

This review verified the synergistic potential of combining the anti-viral therapeutic potential along with the sensitive diagnostic capability of metallic NPs, in a single platform, to manage COVID-19 pandemic. Nanotheranostics is currently regarded as an important tool for the providing both effective therapy and sensitive diagnosis of viral infections in the case of pandemics, therefore more work on the field of nanochemistry is required to synthesize multidirectional nanoparticles that could be employed as multifunctional drug carriers or as sensitive components of detecting and/or imaging systems.

## Data Availability

Not applicable.

## Abbreviations

COVID-19: Coronavirus disease 2019
NPs: Nanoparticles
SARS: Severe acute respiratory syndrome
MERS: Middle East Respiratory Syndrome
WHO: World Health Organization
EM: Electron microscopy
MNPs: Magnetic nanoparticles
AgNPs (SNPs): Silver NPs
AuNPs: Gold NPs
LNPs: Lanthanide-doped polysterene NPs
HCV: Hepatitis C virus
HBV: Hepatitis B virus
FET: Field-effect transistors
TDD: Targeted drug delivery
SeNPs: Selenium NPs
CuNPs: Copper NPs
HIV-1: Human immunodeficiency virus 1
HSV-2: Herpes simplex type 2
GO: Graphene oxide
Po-AuNPs: Porous gold nanoparticles
FCoV: Feline coronavirus
ZnO NPs: Zinc oxide NPs
CeO_2_: Cerium dioxide
IONPs: Iron oxide NPs
SIONPs: Superparamagnetic iron oxide NPs
TiNPs: Titanium nanoparticles
PLGA: Poly lactic-co-glycolic acid
HSV: Herpes simplex virus
AFM: Atomic force microscopy
XPS: Ray Photoelectron Spectroscopy
FESEM: Field emission scanning electron microscopy
LSPR: Localized Surface Plasmon Resonance
FTIR: Fourier Transform Infrared Spectroscopy
EDX: Energy-dispersive X-ray
XRD: X-ray diffraction
TEM: Transmission electron microscopy
SEM: Scanning electron microscopy
ROS: Reactive oxygen species
RNS: Reactive nitrogen species
POC: Point-of-care
